# Classification of Speech and Associated EEG Responses from Normal-Hearing and Cochlear Implant Talkers Using Support Vector Machines

**DOI:** 10.3390/audiolres15060158

**Published:** 2025-11-18

**Authors:** Shruthi Raghavendra, Sungmin Lee, Chin-Tuan Tan

**Affiliations:** 1 Independent Researcher, Santa Clarita, CA 91350, USA; 2 Department of Speech-Language Pathology and Audiology, Tongmyong University, Busan 48520, Republic of Korea; slee18@tu.ac.kr; 3 Department of Electrical and Computer Engineering, The University of Texas at Dallas, Richardson, TX 75080, USA; chin-tuan.tan@utdallas.edu

**Keywords:** cochlear implant, support vector machine, electroencephalogram

## Abstract

**Background/Objectives**: Speech produced by individuals with hearing loss differs notably from that of normal-hearing (NH) individuals. Although cochlear implants (CIs) provide sufficient auditory input to support speech acquisition and control, there remains considerable variability in speech intelligibility among CI users. As a result, speech produced by CI talkers often exhibits distinct acoustic characteristics compared to that of NH individuals. **Methods**: Speech data were obtained from eight cochlear-implant (CI) and eight normal-hearing (NH) talkers, while electroencephalogram (EEG) responses were recorded from 11 NH listeners exposed to the same speech stimuli. Support Vector Machine (SVM) classifiers employing 3-fold cross-validation were evaluated using classification accuracy as the performance metric. This study evaluated the efficacy of Support Vector Machine (SVM) algorithms using four kernel functions (Linear, Polynomial, Gaussian, and Radial Basis Function) to classify speech produced by NH and CI talkers. Six acoustic features—Log Energy, Zero-Crossing Rate (ZCR), Pitch, Linear Predictive Coefficients (LPC), Mel-Frequency Cepstral Coefficients (MFCCs), and Perceptual Linear Predictive Cepstral Coefficients (PLP-CC)—were extracted. These same features were also extracted from electroencephalogram (EEG) recordings of NH listeners who were exposed to the speech stimuli. The EEG analysis leveraged the assumption of quasi-stationarity over short time windows. **Results**: Classification of speech signals using SVMs yielded the highest accuracies of 100% and 94% for the Energy and MFCC features, respectively, using Gaussian and RBF kernels. EEG responses to speech achieved classification accuracies exceeding 70% for ZCR and Pitch features using the same kernels. Other features such as LPC and PLP-CC yielded moderate to low classification performance. **Conclusions**: The results indicate that both speech-derived and EEG-derived features can effectively differentiate between CI and NH talkers. Among the tested kernels, Gaussian and RBF provided superior performance, particularly when using Energy and MFCC features. These findings support the application of SVMs for multimodal classification in hearing research, with potential applications in improving CI speech processing and auditory rehabilitation.

## 1. Introduction

Individuals with severe hearing loss usually lose their ability to produce speech normally. The intelligibility of their speech diminishes over time, which can severely hinder their day-to-day communication. Studies have shown that in children who are prelingually deaf and receive a cochlear implant (CI) at a much younger age, before the age of four, the development of their speech production ability can be comparable to their normal-hearing (NH) peers [1]. However, individual performance with a CI is dependent on various factors, including the age of implantation, duration of hearing loss, the presence of residual hearing, etc. [2,3].

Previous studies in classifying various impairments in the voice such as those due to neurological, traumatic, psychogenic disorders, etc., refs. [4,5,6] commonly use machine learning algorithms on various acoustic features such as Mel-Frequency Cepstral Coefficients (MFCCs), energy and pitch features, etc. The same approach was also employed for word-based and frame-based classification of the speech produced by CI users and hearing-aid users [7]. In our study, we are extending the investigation of the efficacy of one of these machine learning algorithms—the Support Vector Machine (SVM) algorithm, with four different kernel functions—in classifying the continuous speech produced by NH talkers and CI talkers using different acoustic features. The cortical responses to the same speech produced by NH talkers and CI talkers in 11 normal-hearing listeners were recorded using the electroencephalogram (EEG). The feature extraction methodologies used to extract acoustic features were also applied to extract brain wave features from the EEG signal. This is based on the ‘quasi-stationarity’ property observed in EEG signals, which is also observed in speech signals. If the EEG signals are examined over a short period of time (5 to 100 ms), the signal characteristics in EEG are assumed to be stationary. In this paper, we quantified the differences in various acoustic features extracted from the speech produced by NH talkers and CI talkers, as well as the EEG collected in response to the speech produced by the two talker groups from normal-hearing listeners, in an attempt to classify them using SVM.

Recent studies have explored deep-learning frameworks such as CNNs, RNNs, and hybrid SVM–CNN models for EEG-based speech decoding and classification [8,9,10]. These approaches, while they demonstrate promising accuracies, typically require large, balanced datasets and intensive computational resources. Moreover, deep models often lack interpretability and can be prone to overfitting when applied to small-scale neurophysiological datasets. In contrast, traditional kernel-based classifiers such as SVMs have proven advantageous in constrained-data scenarios due to their strong generalization ability, reduced parameter tuning, and robustness to noise. However, previous SVM-based approaches have primarily focused on unimodal speech or EEG signals, with limited work integrating both modalities in a unified analysis. This study addresses this gap by evaluating SVM kernels for combined speech–EEG classification of normal-hearing and cochlear implant talkers. Given the modest data size in this proof-of-concept study, SVMs were chosen to minimize overfitting and to enable systematic comparison across kernel functions. Future work will investigate deep architectures and ensemble approaches once larger, more balanced datasets become available.

The primary contributions of this work are threefold: (1) introducing a multimodal classification framework combining speech and EEG-derived features within a unified SVM-based pipeline, (2) providing a systematic comparison of multiple kernel functions (Linear, Polynomial, Gaussian, RBF) across both modalities, and (3) identifying the most discriminative acoustic and neural features for differentiating cochlear implant and normal-hearing speech. To our knowledge, this is among the first studies to apply SVM kernels simultaneously to speech and EEG data to evaluate auditory–neural correspondence.

The remainder of this manuscript is organized as follows: Section 2 details the acoustic features extracted from speech and EEG signals. Section 3 describes the SVM-based classification model and kernel functions. Section 4 presents the data acquisition and feature extraction procedures. Section 5 discusses the experimental results and analysis, followed by conclusions and future work in Section 6.

### Related Works

Several studies have explored machine learning techniques for classifying speech and EEG responses in normal-hearing (NH) individuals and cochlear implant (CI) users. These works primarily focus on predicting auditory performance, artifact removal, emotion recognition, and speech enhancement using models like Support Vector Machines (SVM), Random Forests (RF), and neural networks. Below, we discuss relevant works in detail, highlighting their techniques, performance, and limitations, drawing from the references in this manuscript.

Seifert et al. (2002) [1] conducted a descriptive analysis of voice and articulation changes in children post-CI. The technique involved qualitative assessment of speech production, with no performance metrics reported (N/A; qualitative; no ML). Limitations include small sample size (n = 10) and absence of machine learning or EEG integration.

Kim et al. (2018) [2] used advanced ML techniques (not specified in abstract) on postlingually deaf adults to predict CI outcomes. Prediction accuracy was not reported in the abstract. Limitations include focus on implantation timing, with no EEG or speech classification.

Ruff et al. (2017) [3] performed acoustic analysis of speech quality versus hearing loss duration in CI users. The approach was descriptive, with no performance metrics (N/A). Limitations include no ML and reliance on clinical correlations only.

Dibazar et al. (2002) [4] applied feature analysis (MFCC, energy) for pathological speech detection. Performance metrics were not reported. Limitations include general voice pathology focus, not CI-specific.

Grygiel et al. (2011) [5] utilized Mel Cepstral processing + SVM for diagnosing vocal disorders from voice recordings. Accuracy was ~80%. Limitations include non-CI population and small dataset.

Pishgar et al. (2018) [6] employed Mel-Cepstrum (MFCC + delta) features with an SVM (RBF kernel) for pathological voice classification, achieving a weighted 5-fold cross-validation score of 0.7469 (≈74.7%). Limitations include focus on general voice disorders—not CI or EEG data.

Mahmoudi et al. (2011) [7] used multiple classifier fusion for voice disorders in children with CI/hearing aids. Accuracy: 96.9% (Avg). Limitations include word-based analysis, no continuous speech or EEG.

Mendel et al. (2017) [11] created a corpus for deaf speech acoustic and production research. No performance metrics (N/A; data collection). Limitations include no classification, focusing solely on acoustic corpus development.

Tan et al. (2015) [12]—Semi-supervised SVM on pre-implant fMRI (speech vs. silence contrast) to predict CI language outcomes; Accuracy = 81.3% (up to 93.8% with 2 features); fMRI-only, no EEG integration.

Raghavendra et al. (2022) [13,14] introduced a single-trial EEG regenerative model for cortical entrainment to CI/NH speech. Correlation: NH = 0.12, CI = 0.08 (*p* < 0.05). This prior work serves as the foundation for the current SVM analysis, extending to multimodal classification.

These studies underscore the novelty of our approach, which is the first to integrate SVM kernels with both continuous speech acoustics and EEG-derived features from the same stimuli, achieving up to 100% accuracy on Energy features. The key findings, methodologies, and limitations of the referenced studies are summarized in Table 1 for clarity and comparison.

## 2. Speech/EEG Features

A set of six acoustic features were chosen, such as the log energy and pitch(f0) to characterize for prosodic aspects of the speech, whereas the linear predictive coefficient (LPC), an articulatory feature, was included to consider the physiological differences in the two groups of speech. MFCCs and perceptual linear predictive cepstral coefficients (PLP-CC) were the perceptual features [15]. Zero-crossing rate (ZCR) was one of the physical features [16] of speech, which was also extracted.

### 2.1. Log Energy

The logarithmic energy is calculated as follows:

(1) $$E={log}_{10}(\sum_{n=1}^{N}s^{2}(n))$$

where *N* represents the number of samples in one frame and *s* is the speech input signal. The frame logarithmic energy is calculated by fragmenting the speech waveform into a number of frames by windowing [17]. All logarithmic computations in this study were performed using log_10_ to maintain consistency across energy and spectral domains, as log_10_ scaling provides normalized magnitudes suitable for comparative feature analysis in both speech and EEG signals.

### 2.2. Zero-Crossing Rate

The ZCR is explained as the rate at which the amplitude of a speech waveform passes the zero-axis at a specific time. ZCR provides information about the signal’s frequency content. Normally, the value of ZCR would be about 14 for the voiced part and about 49 for the unvoiced part in a sample of 10 ms speech data. ZCR is calculated as follows [18]:

(2) $$Z_{n}=\sum_{m=-\infty }^{m=\infty }⎸sgn\left[x\left(m\right)\right]-sgn\left[x\left(m-1\right)\right]⎹\;w(n-m)$$

where

(3) $$sgn\left[x\left(n\right)\right]=\left\{\begin{array}{c} -1,\;\;x(n)<0\\ 1,\;\;x(n)\geq 0 \end{array}\right.$$

and

(4) $$w(n)=\left\{\begin{array}{c} 1/2N,\;\;N-1\geq n\geq 0\\ 0,\;\;otherwise \end{array}\right.$$

where *x*(*n*) is the input speech signal, *w* is the window defined by (4) and *m* represents the frame.

### 2.3. Pitch

Pitch is estimated from the fundamental frequency of the audio signal [18]. The short-term autocorrelation function is used in our study to calculate the pitch of a speech signal, given as follows:

(5) $$R_{n}(k)=\sum_{m=-\infty }^{m=\infty }\left[x\left(m\right)w\left(n-m\right)\right][x\left(m+k\right)w\left(n-m-k\right)]$$

where *x* is the input audio signal, *w* is the window, and *k* represents lag or shift. Pitch was estimated using an autocorrelation-based method with a 25 ms Hamming window and 10 ms step size. The lag search was restricted to 2.5–12.5 ms, corresponding to a pitch range of 80–400 Hz. Unvoiced frames were excluded using an energy-based voicing decision, and pitch outliers deviating by more than two standard deviations from the local mean were removed before feature extraction.

### 2.4. Linear Predictive Coefficients

The notion supporting linear predictive analysis is that speech can be represented by the linear combination of past p speech samples (p-prediction order) [18]. Linear prediction coding is a method of modeling the vocal tract transfer function to estimate formant frequencies [19]. In our study, the autocorrelation formulation was employed to obtain LPC.

### 2.5. Mel-Frequency Cepstral Coefficients

Mel-frequency analysis is a perceptual speech analysis which mimics the functioning of a human ear, which is more sensitive to lower frequencies than to higher frequencies. This can be represented by the Mel-scale which establishes the relationship between the perceived frequency *fmel* and the actual frequency value *flinear*, and it is formulated as follows:

(6) $$f_{mel}=2595\;\times {log}_{10}\left[1+\frac{f_{linear}}{700}\right]$$

**Figure 1:**
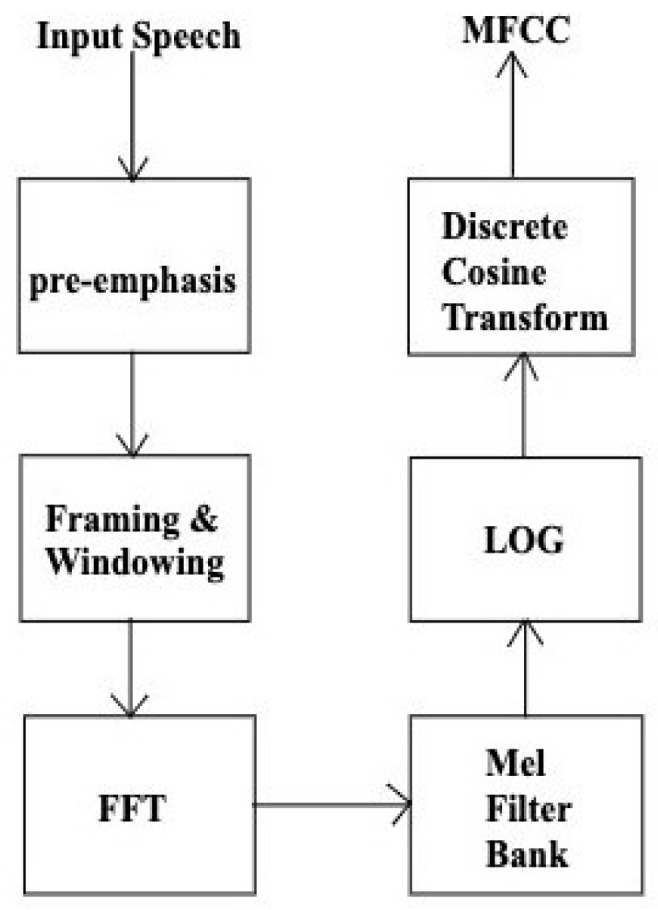
Computational flow chart of MFCC technique.

The flow chart of MFCC computation is shown in Figure 1. Mel-frequency analysis employs a greater number of triangular filters in the lower frequency region and fewer in the higher frequency region along the Mel-scale [20]. The next step is to calculate the log energy of the output of each filter, followed by a discrete cosine transform (DCT) to transfer to the quefrency domain as given by the following:

(7) $$C_{m}=\sum_{k=1}^{N}cos\left[m\times \left(k-0.5\right)\times \frac{\pi }{N}\right]\times x_{k}$$

MFCCs are the output, where *m* = 1, 2, …, L. L is the number of Mel-scale cepstral coefficients and *N* is the number of triangular filters.

### 2.6. Perceptual Linear Predictive Cepstral Coefficients

The perceptual linear predictive (PLP) analysis of speech [21] is based on the perceptual aspect of the human ear. The PLP cepstral coefficient (PLP-CC) computation starts with windowing the speech signal and then calculating the power spectrum as follows:

(8) $$P\left(\omega \right)=Re{\left(S\left(\omega \right)\right)}^{2\;}+Im{\left(S(\omega )\right)}^{2}$$

where *S* is the spectrum of the speech. The PLP feature vector was calculated by the psychophysical transformation of the power spectrum of the speech signal.

## 3. Classification Model

*Support Vector Machine*

**Figure 2:**
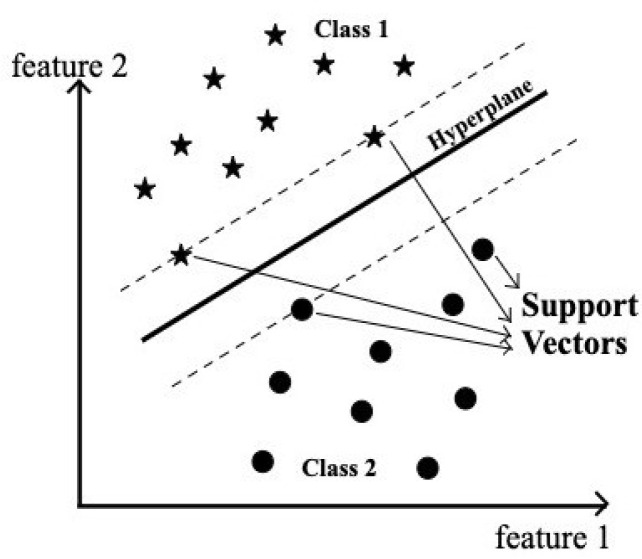
Schematic representation of the Support Vector Machine (SVM) model. The black dots represent data points belonging to Class 2, while the stars represent data points from Class 1. The solid line indicates the optimal separating hyperplane, and the dashed lines denote the margins defined by the support vectors.

Support Vector Machine (SVM) is one of the supervised machine learning algorithms. SVM aims for maximum separation between data points of different classes. Classification is achieved by constructing one or more optimal hyperplanes that achieve a distance that is as far as possible from nearby data points that belong to any class, as shown in Figure 2. The data points lying on the margin on either side of the hyperplane are called support vectors. In our experiment, SVM was employed as a classification algorithm because of its better classification performance compared to any other machine learning algorithms [22]. Hard margin SVM can be applied to linearly separable data as it is rigid and there is no flexibility for any misclassification by the model.

The linear classifier finds the function/hyperplane *f*(*x*) which is given as follows:

(9) $$f\left(x\right)=w^{T}x+b$$

with the largest margin such that sign(*f*(*x*)) = +1, for a positive example and sign(*f*(*x*)) = −1, for a negative example, where w is a weight vector, *x* is the input vector, and b is a bias.

Soft margin SVM can be applied to data points that are not linearly separable by mapping original data into higher dimensional space. This is achieved by using a kernel function between the pairs of all training examples (*x_i_*, *x_j_*) which is given as follows [23]:

(10) $$k(x_{i},x_{j})={\left(x_{i}^{T}x_{j}\right)}^{2}$$

In our paper, we employed four SVM kernel functions to classify the data points:

- Linear kernel,

(11) $$k(x_{i},x_{j})=x_{i}^{T}x_{j}+c$$

- Polynomial kernel (Polynomial degree d),

(12) $$k\left(x_{i},x_{j}\right)={\left(\alpha x_{i}^{T}x_{j}+c\right)}^{d}$$

- Gaussian Kernel,

(13) $$k(x_{i},x_{j})=exp(-\frac{∥x_{i}-x_{j}∥}{2\sigma^{2}}\;)$$

- Radial Basis Function kernel,

(14) $$k(x_{i},x_{j})=exp(-\gamma {∥x_{i}-x_{j}∥}^{2});\;\gamma =\frac{1}{2\sigma^{2}}$$

Slope α and constant *c* are the variables that can be tuned and σ decides the width of the Gaussian kernel.

Linear kernels are faster and can be useful when the data is linearly separable without the necessity of being transferred to a higher-dimensional space. Its most common application is in text classification. Polynomial kernels utilize the correlation between the data vectors and also between the combination of vectors. The Gaussian kernel is one specific example of the Radial Basis Function kernel (RBF). The advantages of the RBF kernel are that its mapping is infinite-dimensional and it results in a non-parametric model, wherein the prediction accuracy increases with the amount of data available for training and testing.

## 4. Materials and Methods

### 4.1. Data Acquisition

Speech: The CI and NH talkers’ recordings were obtained from the Corpus of Deaf Speech for Acoustic and Speech Production Research [11], which remains one of the most comprehensive publicly available corpora featuring synchronized acoustic recordings of cochlear implant users. The dataset was originally collected to study acoustic–phonetic variability and was used here for algorithmic validation rather than population-level generalization. The inclusion of eight CI and eight NH talkers ensured gender balance and covered a broad age range (16–77 years) to capture intra-group variability. While the corpus does not contain details such as months post-implantation or speech recognition scores, it reflects the heterogeneity of the CI population, which was essential to test the algorithm’s robustness against naturally occurring variation. Future work will include expanded datasets with metadata on device type, duration of implant use, and clinical outcomes. The data consists of the recorded speech passages of 8 CI talkers (4 females; 4 males) and 8 NH talkers (6 females; 2 males) reading “The Rainbow Passage”. The CI group comprised individuals between 16 and 77 years of age with severe sensorineural hearing loss with a pure-tone average of at least 70 dB HL (decibel hearing level), whereas the NH group was aged between 15 and 51 and had hearing better than 20 dB HL.

EEG: EEG data were recorded from 11 NH adult listeners who were native speakers of American English, aged from 19 to 29 years (mean age = 21.5 years; 5 female, 6 male) following the protocols approved by the Institutional Review Board at the University of Texas at Dallas. The above speech passages chosen from 8 CI talkers and 8 NH talkers were presented to each NH listener in a randomized order. Their neural responses were recorded using a 64-channel actiCHamp amplifier EEG setup (Brain Products GmbH, Munich, Germany) while listening to the speech passages. Hence, 16 EEG signals were recorded in each listener corresponding to speech passages spoken by 8 CI talkers and 8 NH talkers. The EEG signals were recorded using an electrode cap (actiCAP, Brain Products GmbH, Munich, Germany)) placed in accordance with the 10–20 system [24]. The ground channel and the reference channel were located at FPz and FCz, respectively. All electrode impedances were maintained below 10 kOhms. EEG data were recorded with a sampling rate of 1000 Hz.

All the EEG data were preprocessed using the EEGLAB toolbox [Version14.1.2b] [25] for MATLAB to prune the unwanted artifacts including muscle activity-, eye blinking-, lateral eye movement-, and heartbeat-related artifacts. The EEG preprocessing steps are explained in detail in our previous works [13,14,26].

### 4.2. Feature Extraction

#### 4.2.1. Speech

In our work, Log Energy, MFCCs, and PLP-CC features were the audio features extracted using the open-source Emotion and Affect Recognition toolkit’s feature extracting backend openSMILE [27]. Logarithmic energy was extracted with the cEnergy component in opensmile. Here, the speech was processed frame-by-frame in overlapping intervals, because the speech was considered to be stationary in this short period of time, and the speech features were extracted in those short intervals. The speech signals were analyzed in frames with short window sizes between 20 and 40 ms, with an overlap of 10 ms between frames, so that the signals were assumed to be “quasi-stationary”. In this study, a framing window of 25 ms was considered, with an overlap of 10 ms between frames. For MFCCs and PLP-CC, speech was pre-emphasized with k = 0.97; this process ensured more weight was given to the higher frequencies. Later, MFCCs and PLP-CC features were extracted using a Hamming function after the pre-emphasis step. MFCCs were calculated using openSmile with cMelspec and cMfcc components and PLP-CC from the chain of cFFTmagphase, cMelspec, and cPLP components, which were of 39 and 18 dimensions, respectively, after appending delta and delta–delta coefficients.

ZCR, Pitch, and LPC features were extracted using custom scripts in MATLAB2021b. The LPC spectrum was calculated by the autocorrelation method with a prediction order *p* = 12 using a Hamming window. ZCR and Pitch were extracted by in-built audio extraction functions, zerocrossrate() and pitch(), respectively, in MATLAB2021b.

#### 4.2.2. EEG

The same 6 features were also extracted from EEG signals. To extract Log Energy, MFCCs, and PLP-CC brain wave features, we also used the openSMILE [27] feature extraction toolkit. However, in EEG signal processing, the analysis window size of 25 ms was multiplied by a factor of 10 due to the low-frequency nature of EEG signals, with an overlap of 10 ms between frames. The custom MATLAB scripts used to extract the speech features were also used to extract ZCR, Pitch, and LPC brain wave features. The features were extracted from each of the 64 channels individually, and then features from all 64 channels were merged together.

### 4.3. CI Talker and NH Talker Classification: Speech and EEG

The input feature vector consisted of six features: Energy, ZCR, Pitch, LPC, MFCCs, and PLP. The Polynomial kernel used was of degree 2, as a higher degree would have resulted in an overfitted model. The SVM learner template was created with a different kernel function followed by training a multiclass model as a one-vs-one (OVO) SVM classifier with features and the true labels {1, −1} for NH speech and CI speech, respectively. K-fold cross-validation was performed on the data with k-fold = 3 by dividing the data randomly into 3 subsets of equal size.

Training was performed with 2 (k − 1) subsets and testing/validation with 1 subset with k (=3) iterations to develop the prediction response for testing observations. Accuracy values were calculated and averaged over three runs with training and testing on random data with each feature and with each kernel.

## 5. Results and Discussion

**Figure 3:**
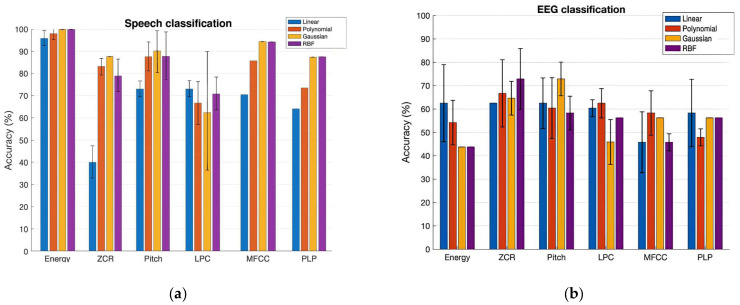
Classification accuracy of normal-hearing (NH) and cochlear implant (CI) talkers using SVM with four kernels. (**a**) Speech features; (**b**) EEG features. The *y*-axis indicates classification accuracy (%), and the *x*-axis lists the six extracted features. Colors correspond to kernel type: blue—Linear, orange—Polynomial, gray—Gaussian, yellow—RBF. Error bars represent ± 1 SD across three independent cross-validation runs.

Talkers were classified using speech: Table 2 and Figure 3a (left panel) show the prediction performance of the SVM classification of NH and CI speech with a set of six features. The bar plot shows the accuracy values of the classification in percentages for the Linear, Polynomial, Gaussian, and RBF Kernels in that order. The variation in the performances of each kernel across three runs is shown in the plot by error bars. Interpreting the results, the Gaussian and RBF kernels both yielded the best accuracy values, achieving 100% with the Energy feature and 94% with the MFCCs feature, respectively. Our experiment’s outcome supports the previous studies, in that the RBF generally outperforms the Linear and Polynomial kernels, as it ensures minimal approximation error in classification problems. This is also true for the Gaussian kernel, since it is one of the forms of RBF kernel.

The better performance of SVM with Log Energy and MFCCs indicates the preservation of the uniqueness of the characteristics of NH speech and CI speech by these two features. Pitch is one of the most commonly considered features in discrimination between normal voices and impaired voices, since it gives a considerable indication of performance, as shown in our study, by the 89% rate of correct classification. We also learned that the ZCR is not a good feature to select to classify NH speech and CI speech; since both are speech signals, they almost have the same rate of sign-changes along the signal, unlike in the case of voiced/unvoiced speech [28] and speech/music [29]. ZCR PLP-CC-based classification gave moderately accurate results. Our findings show that the RBF kernel performs better than the Linear and Polynomial kernels, as the RBF kernel provides infinite-space mapping of the data to classify them. However, considering the tradeoff between the accuracy rate and computational time, the RBF kernel is not recommended for large-scale data.

**Figure 4:**
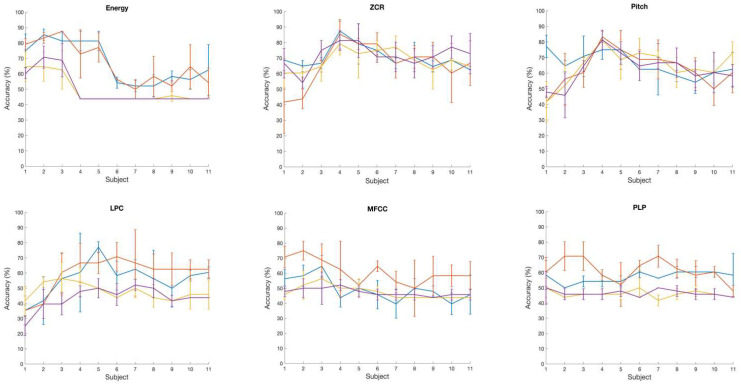
Variation in EEG-based classification accuracy with increasing number of subjects from 1 to 11. Colors represent SVM kernels (Linear = blue, Polynomial = orange, Gaussian = yellow, RBF = purple). Error bars indicate ± 1 SD across three runs.

Talker classification using EEG: Table 3 and Figure 3b (right panel) show the prediction performance of the SVM classification of two talker groups, NH and CI, with a set of six brain wave features. Additionally, RBF and Gaussian kernels both yielded the best accuracy values of 73% with the ZCR and Pitch brain wave features. On the other hand, the Gaussian and RBF kernels performed poorly at classifying the two talker groups with the Log Energy feature. With the LPC, MFCC, and PLP brain wave features, in general, the classification accuracy of all kernels was below 60%. We also analyzed the variation in classification accuracy of the SVM with the increasing number of subjects. At first, only the brain wave features extracted from the EEG of subject 1 were included to evaluate the classification accuracy using four different kernels, followed by adding features from subject 2, subject 3, and so on. Figure 4 shows the variation in accuracy while increasing the number of subjects, showing the calculated average accuracy and also the standard deviation across three runs with six different brain wave features. With the Log Energy, ZCR, Pitch, and MFCC features, a modest increase in classification accuracy was numerically observed when data from approximately four to five subjects were included; however, given the small sample size and the variability across runs, this observation should be interpreted qualitatively rather than as a statistically significant trend. The apparent pattern was derived from mean accuracy values across three cross-validation runs, but no formal statistical testing was performed due to the limited sample size. This observation was not evident with the LPC and PLP brain wave features, for which the variation in accuracy while increasing the number of subjects was almost flat for most of the kernels.

The obtained classification accuracies (up to 100% for speech and 73% for EEG) are comparable to or exceed those reported in previous pathological or auditory classification studies [4,5,7,21]. For example, study [4] reported 97% accuracy in pathological speech detection using MFCC features, while study [7] achieved 87% using hybrid classifiers for CI users. The higher accuracies achieved here can be attributed to feature optimization and careful kernel selection, demonstrating the robustness of SVMs in small-sample multimodal analyses.

Given the limited sample size and the exploratory nature of this proof-of-concept study, formal inferential statistics were not performed. Instead, descriptive statistics (mean ± standard deviation) across three cross-validation runs were reported to indicate the stability of classification performance. The primary purpose of this work was to evaluate the relative trends across features and kernels, rather than to draw population-level statistical inferences.

### Limitations and Future Scope

The current study was limited by the small number of talkers and listeners, which restricts the generalization of the observed classification trends. The CI group also varied in age and device-use history, potentially influencing acoustic variability. Moreover, only six low-level features were examined, without exploring higher-order feature fusion.

Although the sample size was limited, the purpose of this work was to establish proof-of-concept for dual-modality (speech–EEG) classification using identical SVM kernels. Future studies will involve larger CI cohorts with metadata on device configuration and post-implant duration to examine generalizability. Future extensions will include larger datasets, alternative machine learning models such as convolutional neural networks and random forests, and statistical validation with permutation testing to further quantify model robustness.

## 6. Conclusions

This study demonstrated that SVM-based classification can effectively differentiate speech and EEG responses corresponding to normal-hearing and cochlear implant talkers. Among the six examined features, Energy and MFCCs showed the highest discriminative potential for speech, while Pitch and ZCR were the most informative EEG features. The findings highlight the feasibility of integrating acoustic and neural information for multimodal assessment of auditory performance. The conclusion is that Energy and MFCC features capture distinguishing acoustic–phonetic characteristics commonly observed in CI speech—such as reduced spectral contrast, atypical formant structure, and altered prosodic energy distribution—allowing these features to be discriminated from NH speech in the non-linear space. These characteristics likely reflect differences in auditory feedback and speech motor control among CI users, which are known to vary widely across individuals depending on implantation age, device type, and rehabilitation experience.

For EEG-derived features, Pitch and ZCR achieved the highest classification accuracies (~70–72%), demonstrating that cortical responses preserve talker-specific cues. However, limited participant numbers and inter-subject variability constrain generalizability. Future work will expand sample size, include statistical validation, and explore multimodal feature fusion to improve robustness

The results underscore that Gaussian and RBF kernels provide superior non-linear separability in small-sample settings, supporting their use in future auditory classification pipelines. This proof-of-concept framework lays the foundation for neuroacoustic modeling approaches that can guide personalized hearing-aid fitting and cognitive hearing technologies. Future research will extend this model to larger datasets, include feature-level fusion, and evaluate transfer learning for cross-subject generalization.

## Figures and Tables

**Table 1:** Summary of relevant works.

Authors and Year	Techniques Used	Performance Metrics	Limitations/Remarks
Seifert et al. (2002) [1]	Descriptive analysis of voice and articulation changes in children post-CI	N/A (qualitative; no ML)	Small sample (n = 10); no machine learning or EEG
Kim et al. (2018) [2]	Advanced ML (not specified in title) on postlingually deaf adults	Prediction accuracy not reported in abstract	Focus on timing of implantation; no EEG or speech classification
Ruff et al. (2017) [3]	Acoustic analysis of speech quality vs. hearing loss duration	N/A (descriptive)	No ML; clinical correlation only
Dibazar et al. (2002) [4]	Feature analysis (MFCC, energy) for pathological speech detection	Not reported	General voice pathology; not CI-specific
Grygiel et al. (2011) [5]	Mel Cepstral + SVM for vocal disorder diagnosis	Accuracy: ~80%	Non-CI population; small dataset
Pishgar et al. (2018) [6]	Mel-Cepstrum + SVM for pathological voice	Weighted 5-fold CV = 0.7469 (≈74.7%)	General voice disorders; not CI or EEG
Mahmoudi et al. (2011) [7]	Multiple classifier fusion on voice disorders in CI/HA children	Accuracy = 96.9% (Avg)	Word-based; no continuous speech or EEG
Mendel et al. (2017) [11]	Corpus creation for deaf speech analysis	N/A (data collection)	No classification; acoustic corpus only
Tan et al. (2015) [12]	Semi-supervised SVM on pre-implant fMRI	Accuracy: 81.3% (language outcome prediction)	fMRI only, no EEG
Raghavendra et al. (2022) [13,14]	Single-trial EEG regenerative model	Correlation: NH = 0.12, CI = 0.08 (*p* < 0.05)	This study’s prior work—foundation for current SVM analysis

**Table 2:** Average classification accuracies (%) of speech features for normal-hearing (NH) and cochlear implant (CI) talkers using Support Vector Machines (SVMs) with four kernel functions (Linear, Polynomial, Gaussian, and RBF). Gaussian and RBF kernels show superior performance for perceptually relevant features such as Energy and MFCCs.

Feature	Linear	Polynomial	Gaussian	RBF
Energy	97%	99%	100%	100%
ZCR	39%	83%	87%	78%
Pitch	72%	88%	95%	86%
LPC	74%	67%	63%	70%
MFCC	70%	85%	94%	94%
PLP (PLP-CC)	63%	73%	86%	86%

**Table 3:** Average classification accuracies (%) of EEG-derived features for normal-hearing (NH) listeners when exposed to speech from NH and CI talkers. Results are reported for four SVM kernel functions. ZCR and Pitch features exhibit the highest classification performance, particularly with Gaussian and RBF kernels.

Feature	Linear	Polynomial	Gaussian	RBF
Energy	63%	55%	45%	44%
ZCR	63%	68%	72%	71%
Pitch	62%	66%	70%	72%
LPC	63%	58%	64%	61%
MFCC	61%	58%	55%	59%
PLP (PLP-CC)	61%	53%	56%	57%

## Data Availability

The dataset used in this study is not publicly available because it is part of an older project, and the principal investigator (PI) who led the project has since left the institution. As a result, the data has been archived under institutional access controls and is no longer readily accessible for redistribution. Due to these circumstances, we are unable to share the dataset publicly.

## Abbreviations

The following abbreviations are used in this manuscript:

CI	Cochlear Implant
EEG	Electroencephalogram
LPC	Linear Predictive Coefficients
MFCC	Mel-Frequency Cepstral Coefficients
NH	Normal-Hearing
PLP-CC	Perceptual Linear Predictive Cepstral Coefficients
RBF	Radial Basis Function
SVM	Support Vector Machine
ZCR	Zero-Crossing Rate
